# Defining Short-Term Accommodation for Animals

**DOI:** 10.3390/ani13040732

**Published:** 2023-02-17

**Authors:** Clifford Warwick, Catrina Steedman, Mike Jessop, Rachel Grant

**Affiliations:** 1 Emergent Disease Foundation, 71–75 Shelton Street, Covent Garden, London WC2H 9JQ, UK; 2 Veterinary Expert, P.O. Box 575, Swansea SA8 9AW, UK; 3 School of Applied Sciences, London South Bank University, 103 Borough Rd., London SE1 0AA, UK

**Keywords:** animal husbandry, animal accommodation, short-term, temporary, transitional, animal welfare, government guidelines, best practice

## Abstract

**Simple Summary:**

Definitions and usage of the terms short-term, temporary, and transitional are pivotal to animal husbandry and welfare. English Government guidance regarding acceptable short-term, temporary, or transitional accommodation for animals varies widely from <1 day to 3 months; whereas independent scientific criteria and guidance typically use periods of hours to several days. Stipulations regarding acceptable short-term accommodations, notably among English Government guidance, are highly inconsistent and lack scientific rationale. The definitions and use of the terms short-term, temporary, and transitional (for both formal and other guidance) should be limited to precautionary time frames within one circadian cycle, i.e., periods of <24 h. At ≥24 h, all animals at all facilities should be accommodated in conditions that are consistent with long-term housing, husbandry, and best practices.

**Abstract:**

The terms short-term, temporary, and transitional are related but can have different contexts and meanings for animal husbandry. The definitions and use of these terms can be pivotal to animal housing and welfare. We conducted three separate literature searches using Google Scholar for relevant reports regarding short-term, temporary, or transitional animal husbandry, and analysed key publications that stipulate relevant periods of accommodation. English Government guidance regarding acceptable short-term, temporary, or transitional accommodation for animals varies widely from <1 day to 3 months; whereas independent scientific criteria and guidance use typical periods of hours to several days. Stipulations regarding acceptable short-term, temporary, or transitional accommodation, notably among English Government guidance, which we focused on in this study, were highly inconsistent and lacked scientific rationale. The definitions and use of terms for both formal and other guidance should be limited to precautionary time frames within one circadian cycle, i.e., periods of <24 h. At ≥24 h, all animals at all facilities should be accommodated in conditions that are consistent with long-term housing, husbandry, and best practices.

## 1. Introduction

Animals are held captive for various reasons, e.g., as pets or companions, or for zoological, experimental, agricultural, rescue, and other purposes. Modern scientific research and practices unequivocally determine that animals require environments that meet a wide range of criteria, including ethological needs, behavioural choices, and environments that allow them to express a full repertoire of natural behaviours [1,2,3,4,5,6]. Whilst even the best captive environments in the most progressive zoos probably impose lifestyles of controlled deprivation on animals [7], the recognition and aims of keepers for long-term captive care should be to provide conditions that, at the very least, meet essential spatial, climatic, social, behavioural, nutritional, and other needs, as well as additional fundamentals integral to good welfare [8,9,10,11,12,13,14,15]. Inarguably, conditions that fail to meet such essential needs are associated with poor practices and result in reduced animal welfare [14,16,17].

Accordingly, certain conditions are compulsory for meeting essential welfare needs, and typically these conditions are set at standards of compliance with modern science and based on animals’ long-term (rather than temporary) husbandry requirements. Relatedly, in this context, both the husbandry conditions and the length of time during which an animal experiences them can be considered fundamentally relevant to its welfare state. Any situation affecting an animal must aim to maximise its welfare state and minimise negative effects. Therefore, in all situations where higher welfare standards (i.e., best practice conditions for long-term husbandry) are unlikely to be met, such as in short-term or temporary scenarios, protocols must rationally aim to minimise such periods.

### Terminology

The terms ‘short-term’, ‘temporary’, and ‘transitional’ appear frequently in the literature and are applied arbitrarily. This situation leads to both confusion and laxity in their application. Whilst related, these terms can have different implications. For example, using standard Oxford Dictionaries for regular definitions, (1) short-term implies a brief period of time and indicates that change is soon anticipated; (2) temporary may imply brevity or indicate that a situation is impermanent and has an eventual yet open-ended future change; and (3) transitional indicates being in the process of change or transiting. Transportation conditions, whilst also characteristically short-term, temporary, and transitional, are typically considered separately and distinctly from best practice accommodation.

Accordingly, an animal at a veterinary clinic is housed short-term, whereas an animal at a zoo may be on temporary loan for several years, and an animal in a transitional situation may be in the process of being moved around, including transport to onsite or offsite situations. Relatedly, animals transiting via any facility, even when held for an unspecified period of time, could be considered transitional. Most animals at wholesale and retail centres would be considered transitional, regardless of the length of residence, because the intention is to receive or move them onwards. In this context, the term merely reflects temporary practices and cannot be regarded as a determination of what constitutes short-term or long-term animal husbandry. Therefore, the definition and use of ‘short-term’ is pivotal to establishing context and application for other descriptions that have temporal implications for husbandry.

The definition and consistent usage of the term ‘short-term’ would be helpful in coordinating and standardising all regulations involving animal care. Hereinafter, ‘short-term’ can be interpreted to include the terms temporary or transitional. This report aims to present some available information concerning current governmental and scientific guidance regarding the interpretation and application of short-term periods for the confinement of animals, as well as provide objective evidence-led recommendations for both uses of relevant terms and formal and informal policymaking.

## 2. Methods

**Figure 1:**
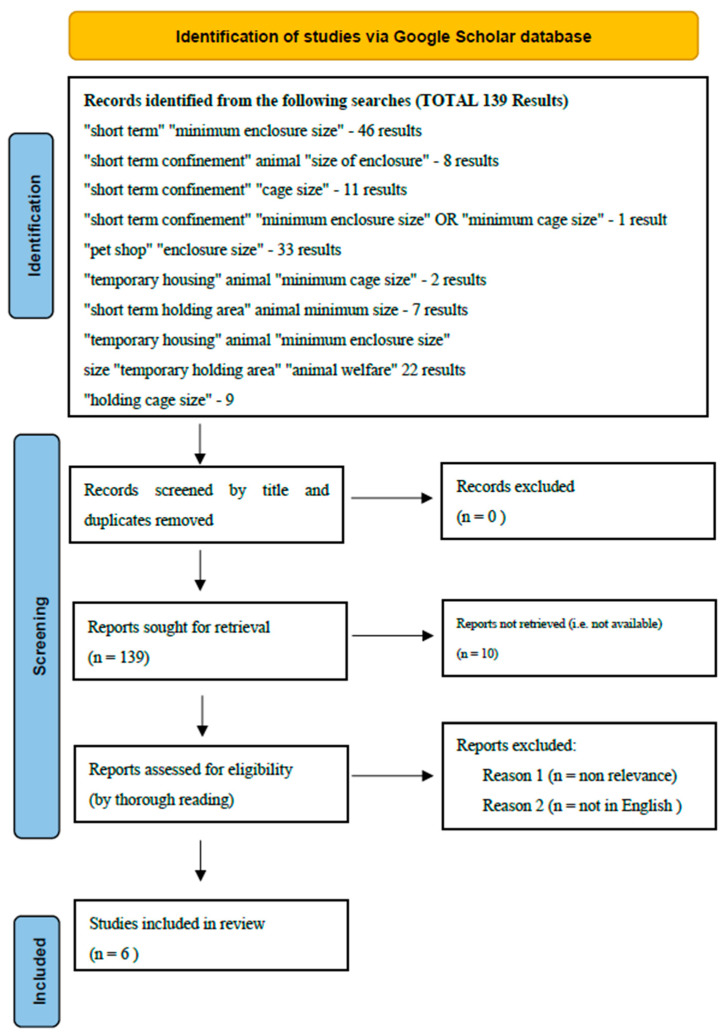
Search terms and strings for short-term, temporary, and transitional accommodation (each search was done separately, the results are combined, and the duplicates are removed). From: Page MJ, McKenzie JE, Bossuyt PM, Boutron I, Hoffmann TC, Mulrow CD, et al. The PRISMA 2020 statement: an updated guideline for reporting systematic reviews. BMJ 2021, 372, n71. doi: 10.1136/bmj.n71. For more information, visit http://www.prisma-statement.org/ (accessed on 30 January 2023).

A provisional search was conducted using the first 10 pages of Google and the terms short term + animal + housing + accommodation + government; temporary + animal + housing + accommodation + government; and transitional + animal + housing + accommodation + government. This provisional search was conducted in order to identify items that were primarily of government origin that may not appear in regular scientific literature searches. This provisional search identified 34 items. Following the exclusion of irrelevant items, 23 publications remained. Next, using the terms and strings presented in the Figure 1 Prisma diagram, we conducted additional separate literature searches in Google Scholar from general to more specific, in order to access the most appropriate parts of the literature. Each search string was entered into Google Scholar separately, and the results were combined. Any duplicates were removed. The abstracts and titles of the articles were read and scored for relevance to the topic. Articles that did not discuss short-term animal accommodation were removed. The numbers of articles shown refer to those remaining after de-duplication and screening for relevance was carried out.

The provisional Google search identified 14 government publications that contained husbandry guidance recommendations for animals, of which, 5 included information specific to short-term accommodation. From the Google Scholar searches, following the analysis of all initial relevant publications and exclusion of non-relevant items, 27 reports were identified for their inclusion of specific references to short-term, temporary, or transitional animal housing. References to short-term, temporary, or transitional animal housing were usually contained as recommendations within governmental guidance, and as incidental descriptions within independent peer-reviewed scientific articles.

We analysed all reports with regard to their status as government guidance or independent scientific projects, and recorded stipulations of short-term, temporary, or transitional determining references, according to animals of relevance, the context of the situation, and the periods interpreted (Table 1 and Table 2). In particular, we used materials published by the English and Welsh Governments to illustrate current formal guidance and associated issues concerning standards stipulated for short-term, temporary, or transitional accommodation of animals in a variety of situations. In this respect, most of the government regulations cited relate to the English Government’s Department of Environment, Food, and Rural Affairs (Defra).

## 3. Results

Table 1 provides summary information concerning animals, contexts, and stipulations regarding short-term confinement contained within governmental legislation or guidance, and Table 2 provides summary information contained within scientific peer-reviewed articles containing criteria or guidance regarding short-term accommodation and husbandry.

**Figure 2:**
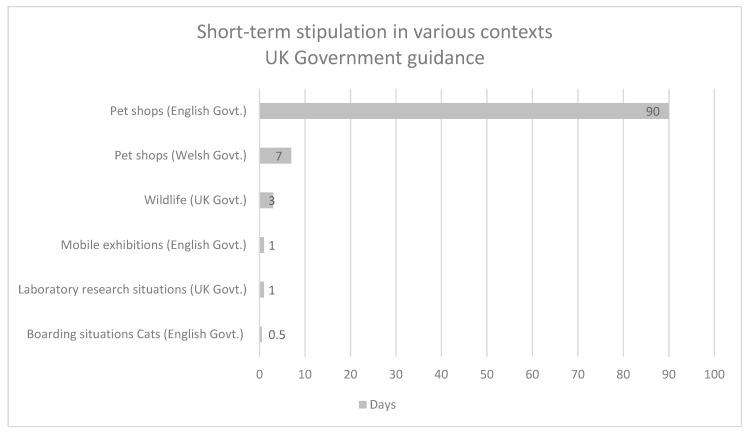
Comparative context for information contained in Table 1. (References [18,19,20,21,22,23]).

**Figure 3:**
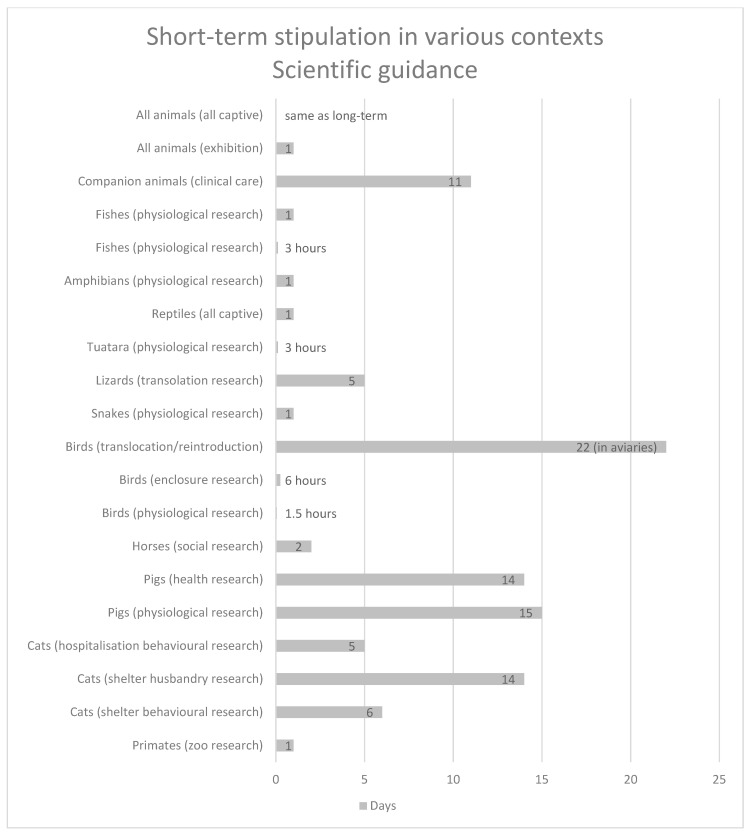
Comparative context for information contained in Table 2. (References [24,25,26,27,28,29,30,31,32,33,34,35,36,37,38,39,40,41,42,43,44]).

Figure 2 and Figure 3 provide a comparative context for information contained in Table 1 and Table 2.

## 4. Discussion

Government guidance information for short-term housing of animals (Table 1) was highly inconsistent, despite largely being produced by the same government department (Defra) in the same year. For example, the two formal guidance documents published by the English Government [18,21] essentially address the same groups of animals under comparable temporary husbandry conditions yet provide widely differing recommendations (short-term = 1 day maximum for holding mobile exhibition animals versus three months for holding animals at retail pet shops). It is worth emphasising that whilst the Welsh Government’s 2021 guidelines [19] adopted most of the English Government’s 2018 guidelines [18], the Welsh Government reduced the short-term stipulation from 3 months to 7 days. A further inconsistency in guidance is that under current English provisions, there are no stipulations at all for animals held at wholesale breeding or supply operations, and again there appears to be no scientific rationale or evidential support bases for this omission.

As a case study, snakes provide a pertinent example of inconsistency in English Government guidance. Under current English Government guidance for pet shops, snakes are the only vertebrate animals that can be kept or sold in conditions where they cannot fully stretch their bodies in accommodation for any defined time period [18]. In comparison, English Government guidance states that all animals (which includes snakes) kept for mobile exhibitions must be able to fully stretch their bodies even under temporary conditions (defined as 1 day) [21]. In contrast, in the Welsh Government’s 2021 [19] reinterpretation of the English Government’s 2018 [18] guidelines, snakes are expected to have the ability to fully stretch under any time-related accommodation. Notably, the English Government has acknowledged that it holds no supporting scientific evidence for this exception affecting snakes, and a formal government agency scientific review of the evidence concluded that snakes should be allowed to fully stretch their bodies [45], which is part of normal health maintenance and good welfare [46,47].

The exceptionally long (three months) definition of temporary or transitional issued by the English Government, solely in respect of animals held at pet shops, was queried with the responsible department (Defra), and formal rationale and supporting scientific evidence were requested. However, no formal response or scientific evidence was provided by the English Government to support its stipulation of three months to constitute temporary or transitional conditions. In comparison, to reiterate, the very similar Welsh Government guidance regulations were amended from stipulating 3 months to 7 days [19]. Manifestly, the English Government’s guidance for short-term accommodation is highly incongruous with normal scientific practices. The English Government has acknowledged prioritising input from vested interests, such as the pet and agricultural industries over independent scientific information, which could in part explain the emergence of arbitrary recommendations that aid some commercial practices rather than reflect scientific validity [48,49,50].

The stipulations for short-term contained within Table 1 represent specific guidance recommendations. However, certain periods and contexts for short-term animal housing contained within Table 2 are less defined and warrant further clarification. Accordingly, reference to ‘short-term captives to have all same benefits as long-term captives’ [24] is included because the original publication indicated that there should be no difference between short- and long-term animal accommodations (i.e., the better environment should be applied); and the reference to ‘long-term conditions applied to short-term housing’ [35] indicated that the study involved the default use of long-term accommodations as part of a short-term project.

### 4.1. The Acute Stress-to-Chronic Stress Transition Paradigm and its Relevance to Short-Term Housing

Stress, defined as a physiological response to potential or actual threats to the organism’s survival [51], is known to affect animals immediately on the impact of a stressor and may have acute or chronic effects [52,53,54]. Acute stress episodes (e.g., social competition, low-level predator-prey interactions, and minor injuries) are usually of short duration, i.e., <30 min [51], and typically fall within the normal coping limits of the individual [55,56,57,58,59], although some severe fearful stress events may produce life-long negative consequences [60]. There are many examples demonstrating how even single short-term stress episodes can have enduring negative consequences. For example, social stress among lizards has been found to impact animals for up to one week [61]. A review of stress (raised corticosterone) in reptiles found the following associations and duration of effects from acute stressors: exposure to salt water 1–4 weeks; social stress (hierarchy with dominating males) = 10–30 days; overcrowding = 10–14 days; and low relative humidity = 3 weeks [62].

Essentially, whilst some acute stress episodes may persist for several days or weeks, other stress-related disturbances to homeostasis caused by acute events can be holistically managed when followed by adequate periods of quiescence and normality. For example, handling an animal may cause it to experience acute stress, but if the animal is then allowed freedom from such interference and able to reside in appropriate shelter conditions, the individual may regain stability within hours or minutes [51,63]. However, at least two overarching considerations are important to this recovery scenario, namely the duration of stressors, and the quality of the recovery conditions.

Continuous stressors (such as invasive light or noise) [64], or multiple acute stress—‘microstress’—episodes [57,58], can result in serious negative health effects, because one episode may effectively rollover to another, resulting in cumulative stress impacts without adequate recovery. Such cumulative impacts from microstressors can facilitate the acute stress-to-chronic stress transition paradigm, resulting in dramatic disturbance of homeostasis and increased morbidity and mortality [54,55,56,57,58,59,65,66,67,68,69,70].

Essentially, all animals are biologically geared within the circadian cycle (a normal 24-h day) [71,72]. In nature, multiple microstressors are conceivable within each circadian cycle. The mechanisms (including physiological and temporal) regarding the transition from acute to chronic stress are not fully understood [72,73]. However, within each circadian cycle, animals naturally experience a normal rest and recovery period within familiar and evolutionarily relevant conditions (e.g., natural habitat, climate, social order) and, thus, such normality aids to facilitate and regain homeostasis [31,72,74]. Acute stressors and their effects that persist beyond a circadian cycle may exceed homeostatic coping limits and can thus be considered to constitute potential chronic stress events by rolling over into the next day, where an animal commences under a pre-existing stress burden [31,63]. Relatedly, disruption of circadian cycles is itself implicated in impaired biological functioning, increased morbidity, and decreased life spans [71,75,76].

An additional issue may be the species-specific speed of life, a factor that considers how a particular length of time may involve disproportionate effects on different species. For example, a circadian cycle may be proportionately (temporally) long for species (or individuals) that are small, short-lived, and have relatively fast metabolic rates compared with the same period applied to species that are large, long-lived, and have relatively slow metabolic rates [77]. Thus, a period of 24 hrs may pass more slowly for some animals, and this potential perceptual duration may be regarded as disproportionately important in a welfare context. Accordingly, and considering all issues thus far mentioned, a precautionary approach that may help to avoid the acute-to-chronic stress transition would be to minimise captivity-associated stressors from persisting beyond 24 hrs.

Moreover, unlike the natural environment, captive enclosures and associated husbandry methods probably involve numerous inherent environmental stressors (e.g., spatial restrictions, under-stimulation, poor social structures, and lack of habitat diversity, as well as abnormal dietary, thermal, lighting, humidity, sound, vibration, and other conditions) that are recognised stressors and could further elicit tipping points from acute-to-chronic stress situations. Accordingly, where single or multiple stressors impact animals in captive environments, the norms that promote homeostasis may be few or absent.

### 4.2. Managing Stressors and Stress

The nervous system of vertebrates detects, processes, integrates, and responds to a variety of external and internal stimuli [78,79]. Processing takes place in the central nervous system and is modulated by a variety of factors, such as age, hormones, and developmental history [78]. After processing, a variety of emergent responses occur that enhance biological fitness. Despite the diversity of species on earth, the foundations of neurobiology (‘afferent—processing—efferent’) are similar across species, as are the ultimate functions of maximising survival and reproduction ([78] p. 928). In addition to this broadly similar basic neurobiology, it must also be recognised that evolution will have selected for particular ‘subcortical neurocircuits’ ([78] p. 928) that produce species-specific physiological and behavioural responses. Learned behaviours will also impact the type, nature, and duration of responses, and indeed can be modified through classical and operant conditioning. Negative emotional states are evolutionarily adaptive, in that they protect the animal from situations that may threaten its survival and/or survival of its offspring [80]. On receipt of a painful or fear-eliciting stimulus animals will either freeze, fight or flee from the perceived danger. The response to stressors (such as fear) arises in two ways.

First, there is a short-term response often called ‘fight or flight’, where catecholamine hormones (dopamine, adrenaline, and noradrenaline) are released from the adrenal glands. These hormones prepare an organism for immediate action by increasing heart rate, widening air passages in the lungs, increasing blood pressure and glucose mobilisation, and numerous other physiological and behavioural effects, to allow the organism to overcome or flee from the dangerous situation [80,81]. The effects of adrenaline/noradrenaline are short-lived, lasting typically minutes to hours, after which the hormones are broken down, the organism’s arousal state returns to baseline, and homeostasis is restored [80]. In the wild, acute stressors can actually improve welfare by inducing appropriate adaptive responses, such as moving away from an aversive stimulus. However, a characteristic of long-term captive situations is the lack of behavioural choice; leading to chronic stress.

Second, there is a longer-term response mediated through glucocorticoid hormones, which take more time to exert their effects, and may have physiological consequences only 20–30 min after the stressful event [81]. If there is no further stressor, through a process of negative feedback, glucocorticoid levels start to drop within an hour; but the effects may last considerably longer [81]. This situation means that animals can recover from acute stressors more readily than chronic stressors. For example, in shelter dogs, cortisol levels were higher after 6 weeks in the shelter than on intake, where intake is likely to have been an acutely stressful event; the long-term kennel environment was clearly a chronic stressor [82]. There is also evidence (e.g., among fishes) that chronic long-term stress reduces the animals’ abilities to cope with subsequent short-term stressors [83]. Therefore, it is important to reduce chronic stress on animals so that they are more resilient to the acute stressors involved in the captive situation, for example, transport, handling, or medical treatments.

Animals occurring within particular sectors, for example, the commercial pet industry, are frequently subject to multifactorial acute and chronic stressors, including wild capture, intensive captive breeding, repeated handling, repeated overly-restrictive confinement and deprivation, local transportation, local transient storage, regional or international transportation, further transient local storage, and other situations (e.g., [84,85,86,87,88]). Accordingly, animals arriving at holding sites may already harbour significant cumulative stress burdens; thus, all efforts should be made to minimise additional negative pressures and affects that would likely derive from subnormal husbandry conditions. Studies show that animals transferred from outdoor to indoor facilities lose condition, likely partly due to increased stressors such as reduced cage sizes [85]. Clearly, animals ought to be provided as soon as possible with environments and protocols that offer the greatest opportunities for rest, recovery, and homeostatic stabilisation, which are implied in relation to environments of long-term best-practice husbandry. Failure to provide such opportunities is arguably tantamount to imposed deprivation and potential harm.

Whilst acute stress should not be an accepted consequence of short-term conditions, short-term housing situations should not cause chronic stress. Any situation that causes chronic (rather than acute) stress should not fall within the definition of short-term or temporary conditions. Short-term conditions should be interpreted as an absolute unavoidable minimum period during which, for overriding practical reasons, environments may not be fully consistent with the Five Freedoms [89], Five Domains [16,17], and other modern principles of welfare. For example, animals presenting at veterinary clinics or rescue facilities, arriving at formal animal import centres, or wholesale or retail pet businesses, may (for essentially brief periods) rationally be held in subnormal environments for specific purposes. Such purposes may include confinement in transportation enclosures, movement between enclosures, or being held in facilities as part of initial processing. Clearly, all such protocols should be pre-planned wherever possible and carried out rapidly in order to ensure that all animals are quickly transferred to higher standard conditions that are fully consistent with long-term husbandry.

## 5. Conclusions

Current terms and practices, relevant to the short-term (or temporary, transitional) housing of animals, whether for pets, display, or agriculture, involve highly inconsistent and largely arbitrary criteria. Such inconsistencies occur even when produced by a single responsible government entity, such as the United Kingdom’s Department of Environment, Food, and Rural Affairs (e.g., stipulations ranging from 12 h in catteries to 3 months in pet shops). Most examples of independent scientific criteria regarding short-term conditions included herein were derived primarily from rationalised research protocols resulting in incidental time scales for confinement, rather than particular recommendations. However, these independent scientific criteria manifestly and consistently adopted typical time periods of hours to several days as scientifically rational stipulations for short-term or temporary conditions of confinement.

Future guidance regarding the definition and use of short-term or temporary conditions requires two essential elements: first, a robust objective scientific rationale; and second, consistent application. Both these elements were lacking in the reviewed English Government and Welsh Government guidance; although in particular regarding information produced by the English Government. We recommend that the definition and use of the term short-term for both formal and other guidance should be limited to periods expressing time frames within the circadian cycle, thus limiting all stipulations in respect of short-term housing to infer periods of 24 h or less. A definition of <24 h for short-term accommodation is practically rational, already in use across governmental guidance, research protocols, and other applied situations, and is entirely generalisable. Moreover, importantly, periods of weeks, or months as stipulated by the English Government for short-term accommodation of animals in pet shops, are at risk of being unenforceable because it may not be possible for regulators to ascertain or validate the actual length of time animals are held within a facility (e.g., due to misplaced paperwork, record-keeping inadequacies, or the absence of individual identification measures). Three months of accommodation in inferior conditions is also clearly inconsistent with good welfare because animals may be subject to greatly prolonged lower standards of care throughout their entire occupation.

## 6. Recommendations

1. The stipulations for short-term, temporary, transitional, or other similarly intended conditions should infer periods of less than a single circadian cycle (typically <24 h).
2. All animals at all facilities should be subject to the single circadian cycle as a principle for determining maximum short-term, temporary, or other transitional conditions.
3. All animals at all facilities must be accommodated in higher or other similarly recognised conditions consistent with long-term husbandry and best practices wherever confinement persists beyond the single circadian principle.
4. Best practice examples of short-term, temporary, or other transitional conditions should include higher standards of husbandry.
5. Keeping animals in short-term, lower standard conditions should be minimised and only for recorded and essential reasons.
6. All animals at all facilities should be subject to government mandatory identification and registration on arrival and departure in order to accurately record their period of stay.

## Figures and Tables

**Table 1:** Short-term (including ‘temporary’ or ‘transitional’) confinement for animals: UK Government legislation or guidance.

Animal(s)	Context	Short-Term Stipulation	Source
All animals	Pet shops	3 months	English Government [18]
All animals	Pet shops	7 days	Welsh Government [19]
All animals	Wildlife	72 h	UK Government [20]
All animals	Mobile exhibitions	24 h	English Government [21]
All animals	Laboratory research situations	<24 h	UK Government [22]
Cats	Boarding situations	12 h	English Government [23]

**Table 2:** Short-term (including ‘temporary’ or ‘transitional’) confinement for animals: scientific criteria or guidance from peer-reviewed academic literature.

Animal(s)	Context	Short-Term Stipulation	Source
All animals	All captive situations	Short-term captives are to have the same benefits as long-term captives	[24]
All animals	Exhibition situations	<24 h	[25]
Companion animals	Clinical care situation	11 h	[26]
Fishes	Physiological research situation	<24 h	[27]
	Physiological research situation	≤3 h	[28]
Amphibians	Physiological research situation	24 h	[29]
Reptiles general	All captive situations	24 h	[30,31]
Tuatara	Physiological research situation	3 h	[32]
Lizards	Translocation research situation	1–5 days	[33]
Snakes	Physiological research situation	1 night	[34]
Birds	Capture and confinement translocation or reintroduction research situation	22 days (in aviaries) = long-term conditions	[35]
	Enclosure research situation	6 h	[36]
	Physiological research situation	<90 min	[37]
Horses	Social research situation	48 h	[38]
Pigs	Health research situation	2 weeks	[39]
	Physiological research situation	1–15 days	[40]
Cats	Hospitalisation behavioural research situation	3–5 days	[41]
	Shelter husbandry research situation	<2 weeks	[42]
	Shelter behavioural research situation	6 days	[43]
Primates	Zoo research situation	1 day	[44]

## Data Availability

Not applicable.
